# Shaping Breast Cancer Treatment in Resource-Limited Settings: Influence of AMAROS and ACOSOG Z0011 Trials in a Follow-up Audit

**DOI:** 10.7759/cureus.94780

**Published:** 2025-10-17

**Authors:** Ammarah Afzal, Muhammad H Nasrullah, Farooq A Rana, Muhammad Saud Iqbal, Najam Us Sahar Hasan, Muhammmad Jawad Haider

**Affiliations:** 1 Surgery, Allama Iqbal Medical College (AIMC), Lahore, PAK; 2 Internal Medicine, Allama Iqbal Medical College (AIMC), Lahore, PAK; 3 General Surgery, Jinnah Hospital Lahore (JHL)/Allama Iqbal Medical College (AIMC), Lahore, PAK; 4 General Practice, Dehli Merchantile Society (DMS) Hospital, Karachi, PAK

**Keywords:** acosog trial, axillary lymph node dissection (alnd), axillary radiotherapy, breast-conserving surgery (bcs), modified radical mastectomy (mrm), sentinel lymph node biopsy (slnb), z0011 trial

## Abstract

Introduction

Trials like AMAROS and ACOSOG Z0011 have modified the approach to sentinel lymph node biopsy (SLNB) in node-positive and node-negative breast cancer. This study evaluates evolving surgical trends in breast cancer management at a resource-limited setting with respect to such trials.

Methods

A follow-up retrospective audit of breast cancer surgeries performed over a three-year period was conducted and compared with the first cycle of audit in the 2017-2021 period. Formal approval was taken from the Ethical Review Board (ERB) of Allama Iqbal Medical College/Jinnah Hospital, Lahore, in its 179th meeting, dated 23-12-2024, with Ref No: ERB179/5(i)/23-12-2024/S1 ERB. Operative records of 273 breast cancer patients treated between 2022 and 2024 were reviewed. Data on age, stage at presentation, and type of surgery were extracted. Surgical procedures included modified radical mastectomy (MRM), breast-conserving surgery (BCS), and SLNB, with or without axillary lymph node dissection (ALND). Analysis was conducted using IBM SPSS Statistics for Windows, Version 27 (Released 2020; IBM Corp., Armonk, New York, United States) to observe trends and compare with the previous audit results.

Results

A majority (70.7%, n=191) of patients presented with Stage III disease, with most cases occurring in the 41-60 age group. Early-stage diagnoses (Stages I and II) increased from 14.2% in the previous audit to 27.9% in this cycle. MRM remained the predominant procedure (72.9%, n=199), though its use declined from 85% in the prior audit to 70% currently. BCS increased from 6% (n=3) in 2022 to 20.3% (n=26) in 2024. Among early-stage patients undergoing BCS with SLNB, 70% (n=28) underwent ALND, while 30% (n=12) did not, reflecting increased adoption of AMAROS and ACOSOG Z0011 guidelines. These trends indicate a progressive shift toward less invasive and guideline-aligned breast cancer management.

Conclusion

The study demonstrates progress in the adoption of conservative breast cancer treatments, despite resource constraints. Emphasis on early detection, adherence to updated guidelines, and periodic audits is critical to improving outcomes in low-resource settings.

## Introduction

Breast cancer remains a major global health challenge, representing the most commonly diagnosed cancer among women and a leading cause of cancer-related mortality. In 2020, approximately 2.3 million new cases and over 685,000 deaths were reported worldwide [1]. Despite medical advancements, significant disparities persist, with nearly 80% of breast cancer-related deaths occurring in low- and middle-income countries (LMICs), primarily due to limited healthcare infrastructure and delays in diagnosis and treatment [2].

While high-income regions have seen increasing breast cancer prevalence, they have also benefited from declining age-standardized mortality rates, largely attributed to improvements in early detection, screening programs, and evolving therapeutic approaches [3]. In contrast, many low-resource settings continue to rely on modified radical mastectomy (MRM) as the mainstay of treatment, often followed by chest wall and regional nodal irradiation [4]. When feasible, breast-conserving surgery (BCS) with negative margins and acceptable cosmetic outcomes should be considered, followed by adjuvant radiotherapy [5]. In this context, periodic institutional audits play a crucial role in evaluating not only treatment patterns but also the degree of progression toward internationally endorsed guideline-based practices.

Over recent decades, management strategies for early-stage breast cancer have increasingly shifted toward less radical and more individualized treatment. Landmark trials such as AMAROS [6] and ACOSOG Z0011 [7] have significantly influenced this paradigm shift, particularly regarding axillary management. The AMAROS trial [6] demonstrated that axillary radiotherapy offers comparable regional control and survival outcomes to axillary dissection, with a lower risk of complications such as lymphedema. Similarly, the ACOSOG Z0011 trial [7] established the safety of omitting axillary lymph node dissection (ALND) in selected patients undergoing BCS, provided only limited sentinel lymph node (SLN) involvement is detected.

Subsequent cohorts have supported these findings, reporting that omitting ALND in patients with ≤2 positive sentinel nodes is both safe and associated with fewer complications, including reduced lymphedema and better arm mobility. The decision to omit ALND may be guided by individual risk profiles, with or without the addition of axillary radiotherapy (ART) [8].

This study aims to conduct a retrospective audit of breast cancer surgeries performed at Jinnah Hospital, Lahore, Pakistan, and compare the findings with a prior audit conducted between 2017 and 2021 [9]. The objective is to assess surgical trends, adherence to evidence-based guidelines, and the influence of institutional practices and resource limitations on treatment decisions. By analyzing three years of surgical data, the study seeks to identify areas of progress and gaps in care, with the goal of informing future strategies for optimizing breast cancer management in resource-constrained environments.

## Materials and methods

A retrospective follow-up study was conducted across all four surgical units of the Department of Surgery at Jinnah Hospital, Lahore, Pakistan. Ethical approval was obtained from the Ethical Review Board (ERB) of Allama Iqbal Medical College / Jinnah Hospital, Lahore, in its 179th meeting held on December 23, 2024 (Ref No: ERB179/5(i)/23-12-2024/S1 ERB).

The study included operative records of breast cancer patients treated surgically between January 1, 2022, and November 1, 2024. All patients with a confirmed diagnosis of breast cancer who underwent surgical intervention during this period were included, regardless of age or clinical stage at presentation. Exclusion criteria comprised incomplete medical or operative records and any history of prior breast surgery before the index admission. This audit involved retrospective analysis of anonymized data. In accordance with institutional ethics policy, no identifiable patient information was used. The Institutional Review Board granted a waiver of individual informed consent due to the non-interventional nature of the study.

Data collection was carried out through manual review of patient records by two trained audit team members over a three-month period, from December 23, 2024, to March 14, 2025. A standardized proforma was used to extract data from the operation theatre registry, supplemented by information retrieved through the hospital’s electronic medical record system (HMIS).

A consecutive sampling technique was employed, whereby all eligible cases within the specified time frame were included. The variables collected included patient age, gender, year of presentation, clinical stage of breast cancer (as documented in medical records), and the type of surgery performed such as MRM, BCS, or sentinel lymph node biopsy (SLNB) with or without ALND. These specific data points were selected as they directly reflect surgical decision-making patterns and guideline adherence, which were the primary quality indicators defined for this audit cycle.

Data were entered and analyzed using IBM SPSS Statistics for Windows, Version 27 (Released 2020; IBM Corp., Armonk, New York, United States). Descriptive statistics were used to summarize demographic and treatment-related variables. Chi-square tests were applied to assess differences in stage distribution and treatment modalities between the two audit cycles. Cochran-Armitage trend tests were employed (see Supplementary Data for analysis tables) to evaluate trends in BCS uptake over time. A p-value <0.05 was considered statistically significant. No pharmaceutical agents or chemical interventions were involved while conducting this study.

## Results

A three-year retrospective analysis of breast cancer surgeries at Jinnah Hospital Lahore identified 273 patients, all of whom were included in the final audit analysis. An additional 24 patients were identified during screening but were not included due to incomplete medical records or a history of prior breast surgery. The most frequent age group was 41-50 years, accounting for 30% (n=82) of the total cohort. Table 1 presents the distribution of clinical stage across different age groups. Notably, a majority of patients presenting with Stage III disease were between 41 and 60 years of age.

**Table 1:** Distribution of Breast Cancer Stages by Age Group Staging based on AJCC TNM classification: Stage I–II: early-stage; Stage III: locally advanced; Stage IV: metastatic disease

Age (years)	I	II	III	IV	Total
<30	3	18	14	1	36
31-40	5	13	33	3	54
41-50	3	14	64	1	82
51-60	3	10	58	2	72
≥61	0	7	24	0	31
Total	14	62	193	7	273

Regarding disease staging at diagnosis, Stage III was most common, observed in 70.7% (n=193) of cases. Stage II accounted for 22.7% (n=62), while early-stage disease (Stages I and II combined) constituted 27.9% (n=76). Advanced-stage presentations (Stages III and IV) made up 73.3% (n=200) of the cohort.

MRM remained the predominant surgical procedure, performed in 72.9% (n=199) of patients. BCS alone was conducted in 12.5% (n=34), while 14.7% (n=40) underwent BCS combined with SLNB. Among patients undergoing BCS + SLN, 70% (n=28) received axillary clearance, whereas 30% (n=12) were managed without axillary dissection in accordance with AMAROS [6] and ACOSOG Z0011 [7] recommendations. Surgical modalities by stage are summarized in Table 2.

**Table 2:** Surgical Approach by Cancer Stage ¹BCS with negative SLN biopsy ²BCS with positive SLN biopsy BCS: Breast-conserving surgery; SLN: sentinel lymph node

STAGE	BCS¹	BCS + SLN²		MRM	Total
		Axillary Clearance Done	Axillary Clearance Not Done		
I	10	3	1	0	14
II	24	25	11	1	61
III	0	0	0	191	191
IV	0	0	0	7	7
Total	34	28	12	199	273

All Stage III cases (n=191) were managed with MRM. In contrast, early-stage cases were more frequently treated with BCS or BCS + SLN, with subsequent axillary clearance or radiotherapy as clinically indicated.

An increasing trend in surgical volume was observed over the study period: 49 surgeries in 2022, 96 in 2023, and 128 in 2024. A corresponding increase in the rate of breast-conserving procedures was also noted, with BCS alone (excluding BCS+SLN cases) accounting for 6% (n=3) in 2022 and rising to 20.3% (n=26) in 2024. These trends are detailed in Table 3.

**Table 3:** Annual Surgical Trends in Breast Cancer Management Note the statistically significant increasing trend in BCS uptake from 2022 to 2024, Cochran-Armitage Trend test (Linear-by-linear association): Z ≈ 3.97, p < 0.001 BCS: Breast-conserving surgery; SLN: sentinel lymph node; MRM: modified radical mastectomy

Year	Treatment Given			Total
	BCS	BCS + SLN	MRM	
2022	3	10	36	49
2023	5	15	76	96
2024	26	15	87	128
Total	34	40	199	273

A gradual increase in guideline-aligned breast-conserving procedures was noted across the audit period, indicating progressive institutional adherence to AMAROS and ACOSOG Z0011 recommendations rather than a change driven solely by surgical volume.

## Discussion

**Figure 1:**
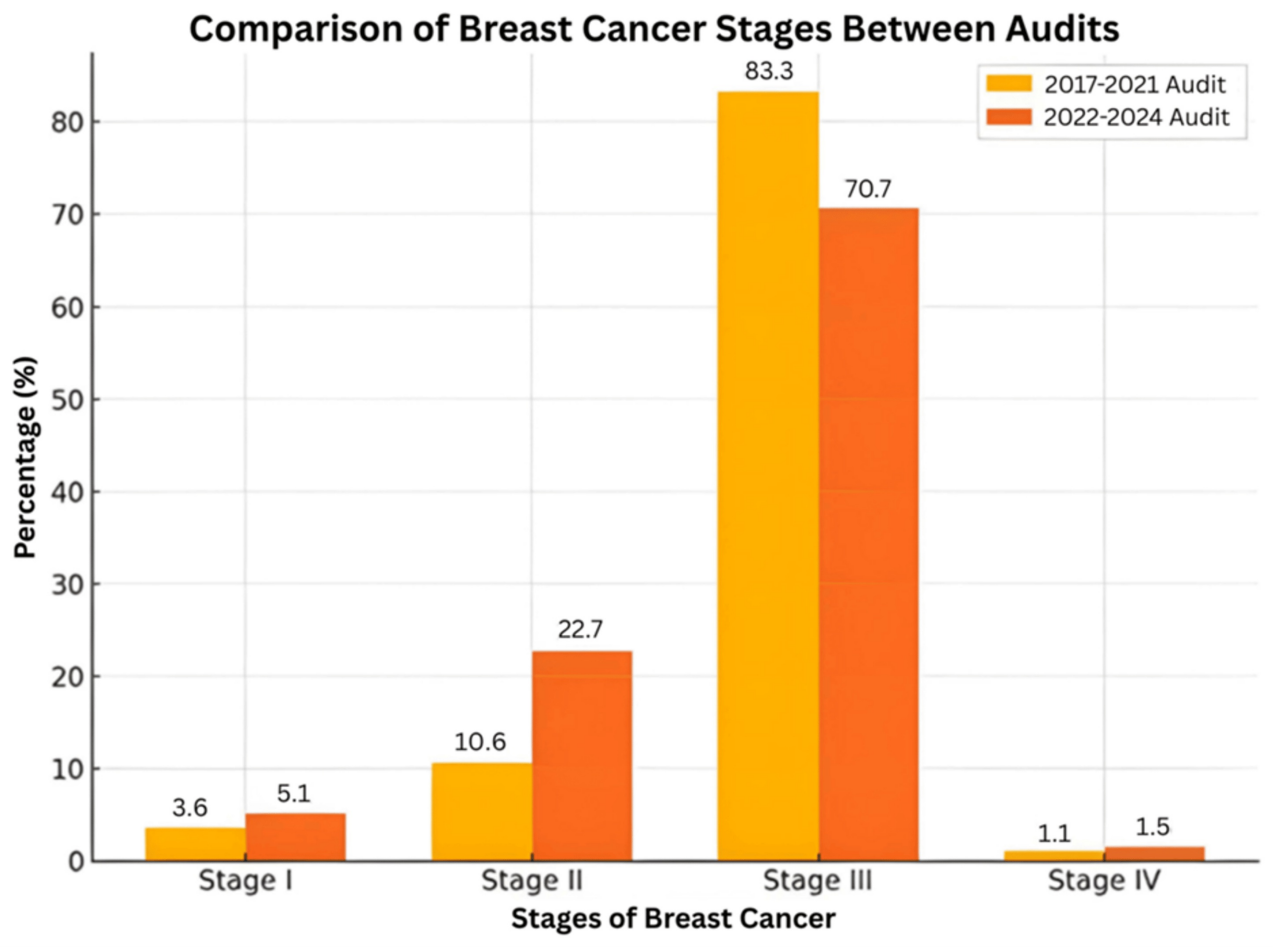
Comparison of Breast Cancer Stages Between Audits The bar graph shows the percentage distribution of breast cancer stages in the 2017–2021 (yellow) and 2022– 2024 (orange) audits. The statistically significant difference in stage distribution between audit cycles indicates a shift toward earlier-stage diagnosis. Chi-square test result: χ²(3) ≈ 31.2, p < 0.001

The comparison between the 2017 and 2021 [9] and 2022 and 2024 audit cycles reveals notable improvements in breast cancer detection and treatment strategies at our institution. As shown in Figure 1, there has been a shift toward earlier-stage diagnosis. The proportion of patients presenting with Stage I disease increased from 3.6% (n=17) to 5.1% (n=14), and Stage II cases rose from 10.6% (n=50) to 22.7% (n=61). A significant difference was observed in cancer staging between the two audit cycles (χ²=31.2, p<0.001), with a shift toward earlier-stage diagnoses in the 2022-2024 cycle. These trends suggest the positive impact of improved screening practices, greater public awareness, and increased access to diagnostic services. However, systemic barriers persist. Studies have shown that challenges such as limited geographic access, diagnostic delays, and long waiting times continue to hinder timely breast cancer care, particularly in LMICs in Asia [10]. Addressing these issues requires targeted investment in healthcare infrastructure, better training of healthcare providers, and expanded diagnostic capacity.

There was a corresponding decline in Stage III cases from 84.5% (n=398) to 70.7% (n=191), which is encouraging. However, a slight rise in Stage IV presentations from 1.3% (n=6) to 2.6% (n=7) highlights the continued need to reach populations that may still be missing early detection opportunities.

**Figure 2:**
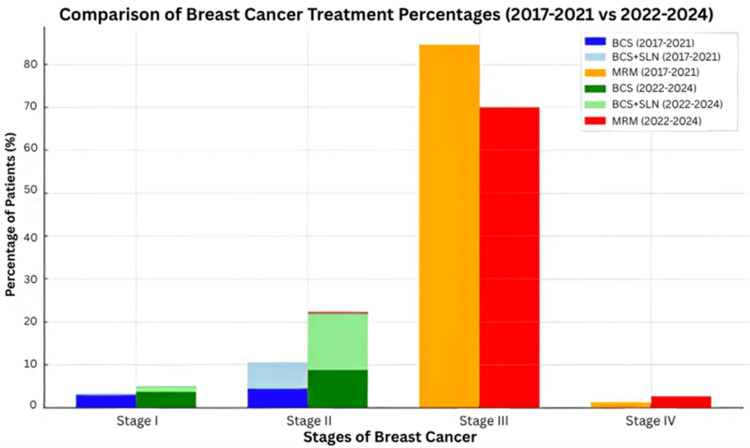
Comparison of Breast Cancer Treatment Percentages (2017– 2021 vs 2022–2024) Significant increase in breast-conserving procedures and corresponding decline in MRM over the years. Chi-square test result: χ²(2) ≈ 52.9, p < 0.001 BCS: Breast-conserving surgery; SLN: sentinel lymph node; MRM: modified radical mastectomy

Surgical trends also demonstrate a gradual move toward less invasive approaches. As shown in Figure 2, BCS in Stage I cases increased from 3% (n=14) in the 2017 to 2021 audit to 4% (n=10) in the 2022 to 2024 cycle. Additionally, BCS combined with SLN biopsy accounted for 2% (n=4). In Stage II, BCS rose from 5% (n=21) to 8% (n=24), while BCS plus SLN increased from 9% (n=29) to 12% (n=36). These changes are consistent with the findings of the AMAROS [6] and ACOSOG Z0011 [7] trials, which support conservative management in select patients. The AMAROS trial demonstrated that axillary radiotherapy can be an effective alternative to ALND in terms of survival and regional control. The ACOSOG Z0011 trial showed that ALND may be safely omitted in patients undergoing BCS who have up to two positive sentinel lymph nodes.

Clinical guidelines from St. Gallen, NCCN, and ASCO also recommend omitting ALND in patients who meet the ACOSOG Z0011 eligibility criteria [7]. This may extend to selected mastectomy patients with one or two positive sentinel nodes who require chest wall radiotherapy, in which case axillary radiotherapy may be offered in place of ALND [11]. Some studies suggest that applying Z0011-based criteria could reduce ALND rates by up to 20 percent [12]. Although MRM remains the most common treatment for Stage III disease, its use has declined from 85% (n=398) to 70% (n=191), indicating a broader acceptance of more conservative practices where appropriate. For Stage IV cases, a modest increase in MRM from 1.3% (n=6) to 2.6% (n=7) may reflect more aggressive treatment approaches in advanced disease. Overall, surgical management patterns showed a significant increase in breast-conserving procedures (BCS and BCS+SLN) and a reduction in MRM (χ²=52.9, p<0.001).

**Figure 3:**
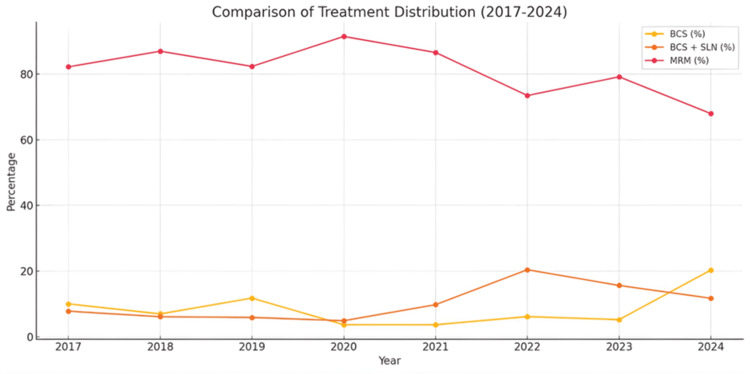
Comparison of Treatment Distribution (2017–2024) The line graph depicts trends in breast cancer treatment over time, showing the percentage of patients undergoing breast-conserving surgery (BCS), BCS with sentinel lymph node biopsy (BCS+SLN), and modified radical mastectomy (MRM). While MRM remains the predominant treatment, a gradual increase in BCS and BCS+SLN is observed, indicating a shift towards less invasive surgical approaches.

A year-by-year comparison, shown in Figure 3, demonstrates evolving trends in surgical practice. From 2017 to 2023, BCS rates remained low, ranging between 3% and 5% (n=8), while the use of BCS with SLN gradually increased, reaching 15.6% (n=15) in 2023. A notable shift occurred in 2024, when BCS alone increased to 20.3% (n=26). A year-wise trend analysis using the Cochran-Armitage Trend test (linear-by-linear association) from 2022 to 2024 demonstrated a statistically significant rise in BCS uptake (Z=3.97, p<0.001), reflecting progressive adaptation of conservative treatment approaches. This may reflect greater alignment with evidence-based protocols and institutional adoption of recommendations from trials such as AMAROS and ACOSOG Z0011. Despite this trend, MRM continues to dominate surgical treatment across all years. One contributing factor may be patient preference. Studies have shown that fear of recurrence is a common reason why women opt for mastectomy rather than breast conservation [13].

Several institutional and contextual factors appear to have influenced the observed surgical shifts toward breast-conserving approaches in the current audit cycle. The increased adoption of guidelines from landmark trials such as AMAROS and ACOSOG Z0011 likely played a pivotal role in shaping clinical decisions, as reflected by the 30% omission rate of ALND among SLN-positive patients. Institutional learning from the previous audit may have enhanced awareness and confidence among surgeons regarding conservative management, while rising surgical volumes from 49 in 2022 to 128 in 2024 suggest improvements in surgical infrastructure and capacity. However, persistent resource limitations, including the need for radiotherapy facilities and longer operative times associated with BCS, continue to restrict its wider implementation, especially in Stage III disease. Cultural factors such as fear of recurrence may also lead patients to opt for MRM despite eligibility for BCS. Collectively, these factors indicate a gradual but meaningful transition influenced by evidence-based training, infrastructural adaptation, and evolving patient and provider preferences within the constraints of a low-resource healthcare setting.

These findings underscore the influence of institutional protocols, national awareness programs, and multidisciplinary approaches on surgical decision-making. Earlier diagnosis and conservative surgical options are associated with improved patient outcomes and reduced morbidity. Furthermore, in low-resource settings, the financial benefits of adopting AMAROS and Z0011 guidelines are significant, as they may reduce the need for more extensive procedures, lower complication rates, and decrease hospitalization costs [14]. For broader implementation, additional policy measures should support guideline dissemination and training.

However, this study has limitations. It lacks data on post-surgical complications, recurrence rates, and follow-up pathology. These limitations highlight the need for future prospective studies that evaluate long-term outcomes, complication rates, and compliance with follow-up protocols. Future research should also examine patient understanding of treatment options and address remaining disparities in early detection and access to care.

Lastly, upcoming reviews should integrate findings from recent trials involving patients with three or more positive lymph nodes [15], as these were not included in the original ACOSOG Z0011 trial population [7].

## Conclusions

This audit highlights a positive transition in breast cancer management within a resource-limited setting, marked by earlier detection and increased adoption of breast-conserving procedures. The reduction in late-stage presentations and the rising use of SLN biopsy in selected patients indicate improved diagnostic capabilities and adherence to evidence-based surgical guidelines. While MRM remains the predominant approach, particularly for advanced disease, its declining frequency reflects a gradual shift toward less invasive strategies. However, persistent challenges such as limited healthcare access, delayed diagnosis, and patient apprehensions continue to affect treatment outcomes. Strengthening health systems, promoting timely diagnosis, and enhancing awareness of guideline-based options are essential steps toward equitable and improved breast cancer care.
